# Emphysematous pyelonephritis with extensive gas accumulations in a patient with poorly controlled diabetes: A case report

**DOI:** 10.1097/MD.0000000000047199

**Published:** 2026-01-16

**Authors:** Chenda Feng, Wenjing Tang, Lichao Sun, Xiaoming Jiang, Rui Kuang, Xinyi Zhao, Jingchun Han

**Affiliations:** a Department of Emergency Medicine, The First Hospital of Jilin University, Changchun, Jilin, People’s Republic of China; b Department of Emergency Critical Care Center, Beijing Anzhen Hospital Affiliated to Capital Medical University, Beijing, People’s Republic of China; c Department of Ultrasound, The First Hospital of Jilin University, Changchun, Jilin, People’s Republic of China.

**Keywords:** antibiotics, diabetes, emphysematous pyelonephritis, percutaneous drainage

## Abstract

**Rationale:**

Emphysematous pyelonephritis (EPN) is a rare but severe necrotic renal parenchymal infection with gas accumulation in the renal or extrarenal spaces. Patient could present as sepsis with a high mortality. Prompt diagnosis and treatments are essential to save the lives of affected patients.

**Patient concerns:**

Here, we report a 64-year-old woman with poorly controlled diabetes. She presented with fever, progressive right flank pain, dysuria, urinary frequency, and urgency.

**Diagnoses:**

A computed tomography scan showed right renal parenchymal necrosis with extensive perinephric gas accumulation that extended inferiorly to the right ureter, bladder, and right iliac fossa, posteriorly to the bilateral psoas and erector spinae muscles, and superiorly to the perihepatic space, mediastinum, left shoulder, and left side of the neck. The massive extent of the gas accumulation was rarely reported previously. EPN was diagnosed.

**Interventions:**

She received intravenous antibiotics, blood glucose control and percutaneous drainage.

**Outcomes:**

She successfully recovered after a series of treatments.

**Lessons:**

Early identification and initiation of etiological treatment, as well as symptomatic treatment for EPN are crucial for saving patients’ lives.

## 1. Introduction

Emphysematous pyelonephritis (EPN) is a rare but severe necrotic renal parenchymal infection with gas accumulation in the renal or extrarenal spaces.^[1]^ Patient could present as sepsis with a high mortality.^[2]^ Majority of the patients had a history of diabetes. In addition, urinary tract obstruction and immunocompromised status could also increase the risk of EPN.^[3]^ The most common pathogen is *Escherichia coli*.^[4]^ Affected patients require immediate systemic antibiotic treatments and fluid resuscitation. Some patients might even require surgical interventions, such as percutaneous drainage or nephrectomy.^[2]^ Here, we report a patient with EPN and extensive gas accumulation who was successfully treated with intravenous antibiotics, blood glucose control, and percutaneous drainage.

## 2. Case presentation

A 64-year-old female presented to the hospital with a fever and progressive right flank pain for 7 days. She also reported dysuria, increased urinary frequency, and urgency for 1 day. Her medical history included diabetes with poorly controlled blood glucose levels. She received unknown oral antibiotics from an outside hospital with no improvements. During the presentation, her vital signs were: temperature 37.5°C, respiratory rate 25 breaths/min, pulse 90 beats/min, and blood pressure 103/52 mm Hg. She was awake but slightly lethargic. Physical examinations were unremarkable except for the percussion tenderness at the right costovertebral angle area.

## 3. Laboratory tests

Routine complete blood count showed white blood cell count 22.4 × 10^9^/L, neutrophil percentage 95%, hemoglobin level 114 g/L, and platelet count 15 × 10^9^/L. Blood chemistry tests showed glucose 14.8 mmol/L, potassium 2.9 mmol/L, sodium 122.3 mmol/L, chloride 87 mmol/L, blood urea nitrogen 43.6 mmol/L, creatinine 274.4 μmol/L, procalcitonin 8.2 ng/mL, and hemoglobin A1C 12.7%. Blood gas analysis reported pH 7.41, PaCO_2_ 32 mm Hg, bicarbonate 20.3 mmol/L, and lactate 1.1 mmol/L. Urinalysis showed positive results for white blood cells, red blood cells, protein, nitrites, and bacteria.

## 4. Imaging tests

**Figure 1.**
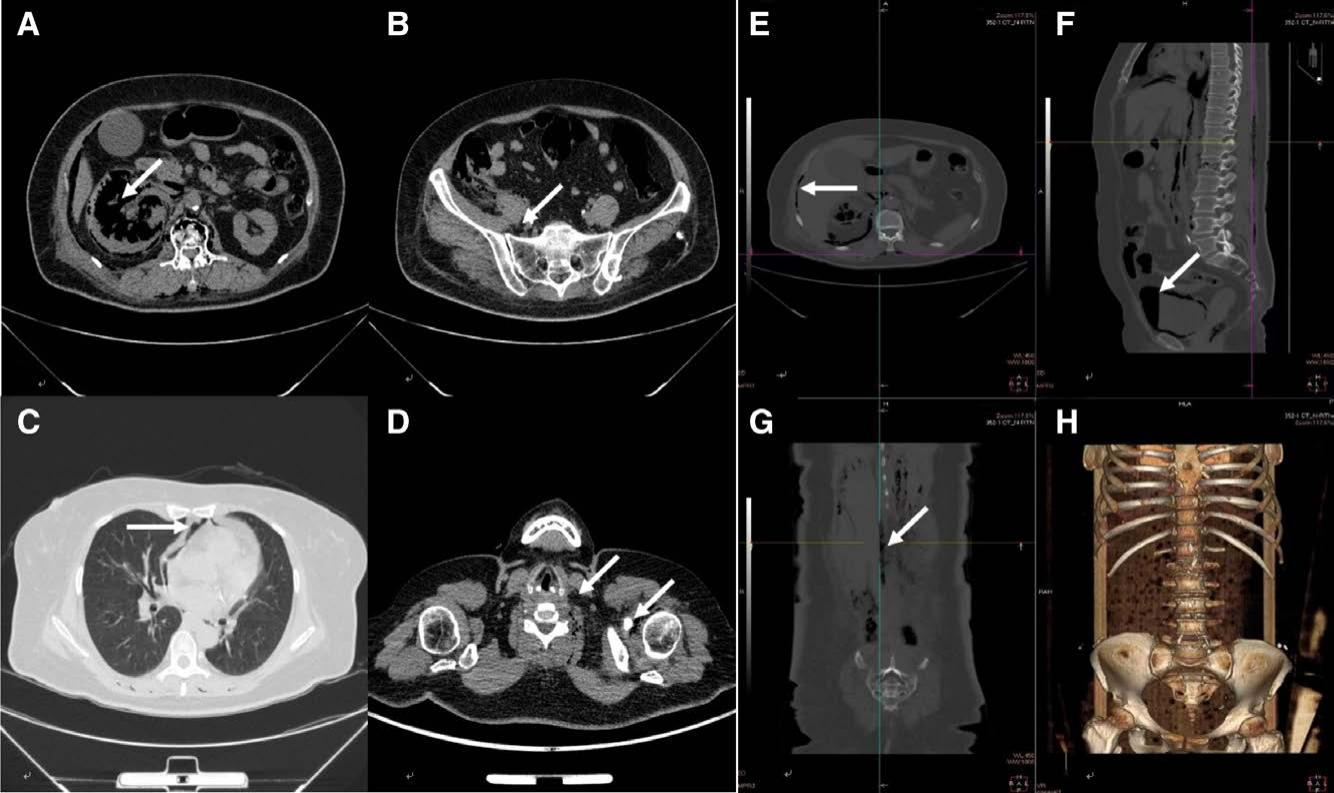
Extensive gas accumulations. Non-contrast-enhanced computed tomography scan shows the right renal parenchymal necrosis, with extensive perinephric gas accumulation (A), extending to involve the right iliac fossa (B), mediastinum (C), left shoulder and left side of the neck (D), perihepatic space (E), bladder (F), bilateral psoas and erector spinae muscles (G), and Extensive pneumoperitoneum and pneumopelvis are identified on imaging (H).

A non-contrast-enhanced computed tomography (CT) scan was performed emergently (Fig. 1). It showed the right renal parenchymal necrosis with extensive perinephric gas accumulation. The gas was extended inferiorly to the right ureter, bladder, and right iliac fossa; posteriorly to the bilateral psoas and erector spinae muscles; and superiorly to the perihepatic space, mediastinum, left shoulder, and left side of the neck.

## 5. Diagnoses

She was diagnosed with EPN, sepsis, acute kidney injury, type II diabetes with hyperglycemia, and electrolyte imbalance.

## 6. Course of treatment

After being sent for blood and urine cultures, intravenous meropenem was started at a dose of 1 g 3 times daily. Blood sugar was controlled by injections of insulin Novocain (30R) 26 units in the morning and 24 units in the evening.

Meanwhile, emergent ultrasound-guided percutaneous renal puncture and drainage were performed with 500 mL of bloody pus aspirated. Finally, the blood culture was positive for *E coli*. After the intravenous antibiotic treatment and percutaneous drainage for 10 days, the patient reported significant improvements with no fever or flank pain. The drainage catheter was removed. Repeat laboratory tests were within the normal limits. Repeat CT scans also showed improvement in renal inflammation and perirenal, bladder and spinal gas accumulation. The patient’s condition gradually stabilized during the treatment period, with no adverse drug reactions and related complications.

## 7. Follow-up results

The patient was discharged from the hospital and followed up in the clinic.

## 8. Discussion

EPN is a severe necrotic renal parenchymal infection. It often occurs in patients with poorly controlled diabetes, urinary tract obstruction, or immunocompromised status. The disease course can be fulminating. The mortality rate could be up to 50%.^[5]^ Moreover, the most common risk factor for EPN is diabetes (75–96%).^[6,7]^ Patients with EPN also have high average hemoglobin A1c level (9.2%). Glucosuria feeds the growth of glucose-fermenting microorganisms, eventually leading to necrotizing infections and gas formation.^[8]^ The most common pathogen is *E coli*. However, infections due to *Klebsiella pneumoniae*, Proteus, *Pseudomonas aeruginosa*, and Candida have also been reported.^[9]^ These infections damaged the renal structure and induced gas production from glucose fermentation and tissue necrosis. The diagnosis of EPN depends on laboratory tests to look for signs of infection and radiographic imaging to examine the renal parenchyma and its surrounding tissue. The non-contrast-enhanced CT scan is usually considered as the image study of choice.^[10]^ The renal parenchymal destruction with gas accumulations are the characteristic CT features. Ultrasound cannot diagnose EPN due to its insensitivity to detect the gas, although it can reveal potential urinary tract obstruction.^[11]^ EPN treatments include broad-spectrum antibiotics, perinephric drainage, and nephrectomy.^[12-14]^

In the present study, we reported a patient with EPN who had extensive perinephric gas accumulation that extended inferiorly to the right ureter, bladder, and right iliac fossa; posteriorly to the bilateral psoas and erector spinae muscles; and superiorly to the perihepatic space, mediastinum, left shoulder, and left side of the neck. Such a massive extent of severe gas accumulation was rarely reported in the previous literature. Our patient had typical clinical presentations of the upper urinary tract infection, with fever, flank pain, dysuria, increased urinary frequency, urgency, and percussion tenderness at the costovertebral angle area. Her noncompliance with the diabetic treatment and hyperglycemia placed her at a high risk of developing a severe infection. Initially, she received unknown oral antibiotic treatment with no improvements. After admitting her into our hospital, in addition to intravenous meropenem, we also tightly controlled her blood glucose level with insulin administration and performed emergent ultrasound-guided percutaneous renal puncture and drainage. She had a satisfactory recovery. Our present case report suggested that, in addition to antibiotic administration in patients with urinary tract infection and poorly controlled diabetes, renal images should be considered, and blood glucose should be treated. If patients show unsatisfactory responses to the initial treatment, more aggressive management should be considered, such as percutaneous drainage or even nephrectomy.

## 9. Conclusion

In summary, EPN is a rare but life-threatening severe infection. In patients with upper urinary tract infection and poorly controlled diabetes, appropriate kidney imaging should be performed. Once EPN is diagnosed, systemic antibiotics should be administrated immediately. In addition, blood glucose level and electrolyte balance should be monitored. Surgical intervention should be considered in EPN patients with a poor response to the antibiotic treatment.

## Acknowledgments

We would like to express our sincere gratitude to the patient for his support.

## Author contributions

**Conceptualization:** Lichao Sun.

**Data curation:** Xiaoming Jiang, Xinyi Zhao.

**Formal analysis:** Rui Kuang.

**Methodology:** Wenjing Tang.

**Supervision:** Jingchun Han.

**Writing** – **original draft:** Chenda Feng.
